# Supplementary material for: Sub-neutralizing concentrations of Zika virus IgM monoclonal antibodies reduce IgG-mediated antibody-dependent enhancement of infectivity
Source: bioRxiv. 2026 Jan 3:2026.01.03.697490. Preprint. [Version 1] doi: 10.64898/2026.01.03.697490 (PMC12930443; doi:10.64898/2026.01.03.697490)
Supplement: Supplement 1 [file media-1.pdf]

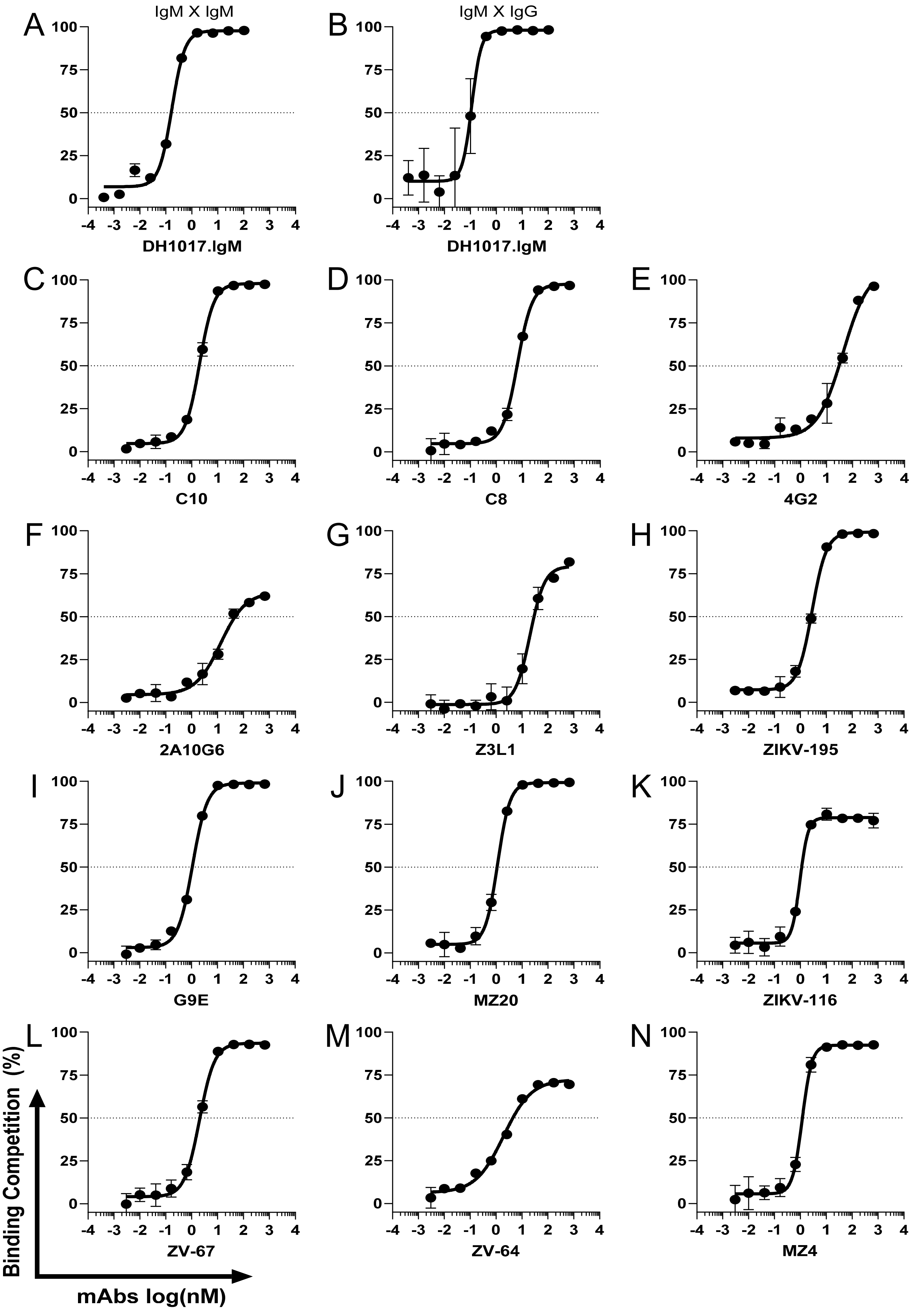
IgM X IgM
IgM X IgG
A
B
C
D
E
F
G
H
I
J
K
L
M
N
DH1017.IgM
DH1017.IgM
C10
C8
4G2
2A10G6
Z3L1
ZIKV-195
G9E
MZ20
ZIKV-116
ZV-67
ZV-64
MZ4
Binding Competition (%)
mAbs log(nM)

Figure S1. ZIKV-DAK whole virion binding autologous competition of DH1017.IgM and ZIKV-IgG mAbs. (**A and B)** Validation of the ability of unconjugated DH1017.IgM to compete binding to ZIKV-DAK whole virions by its own HRP-conjugated versions expressed as an IgM (A) or as an IgG (B). **(C-N)**: Validation of the ability of each unconjugated ZIKV-IgG mAbs to compete binding of their own HRP-conjugated versions. Dotted lines: 50% binding reduction of HRP-conjugated mAbs alone. Error bars: SD of two technical replicates. Data are representative of at least two independent experiments.

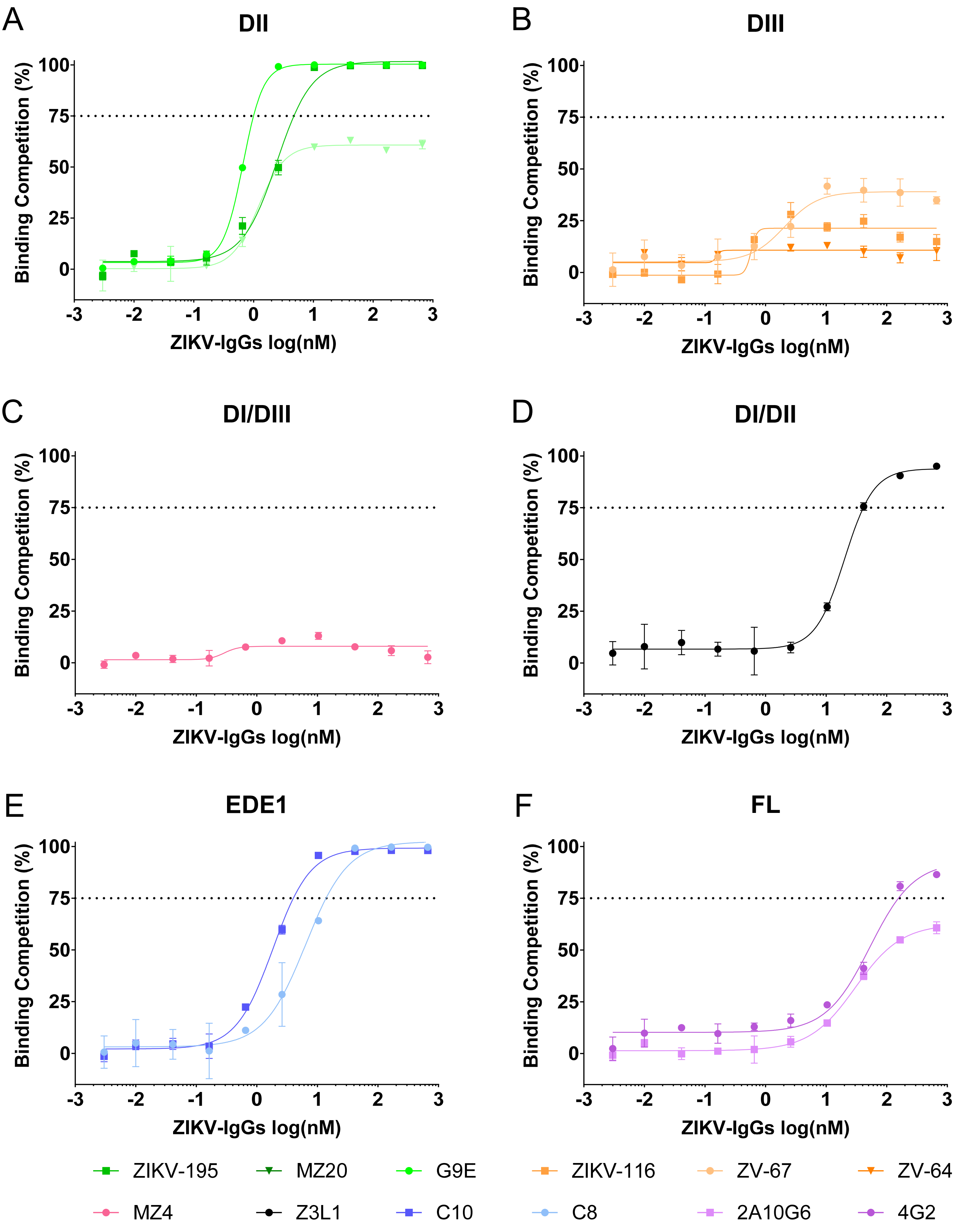

Figure S2. ZIKV-IgG dose-dependent competition of DH1017.IgM binding to ZIKV-DAK whole virion. Dose-response curves show the percentage of DH1017.IgM binding to ZIKV-DAK competed by each of the 12 ZIKV-IgG mAbs grouped in panels based on binding regions: **(A)** DII, **(B)** DIII, **(C)** DI/DIII linker, **(D)** DI/DII hinge, **(E)** EDE1, and **(F)** fusion loop (FL). Dotted lines: 75% binding reduction of DH1017.IgM binding. Error bars: SD of two technical replicates. Data are representative of two independent experiments.

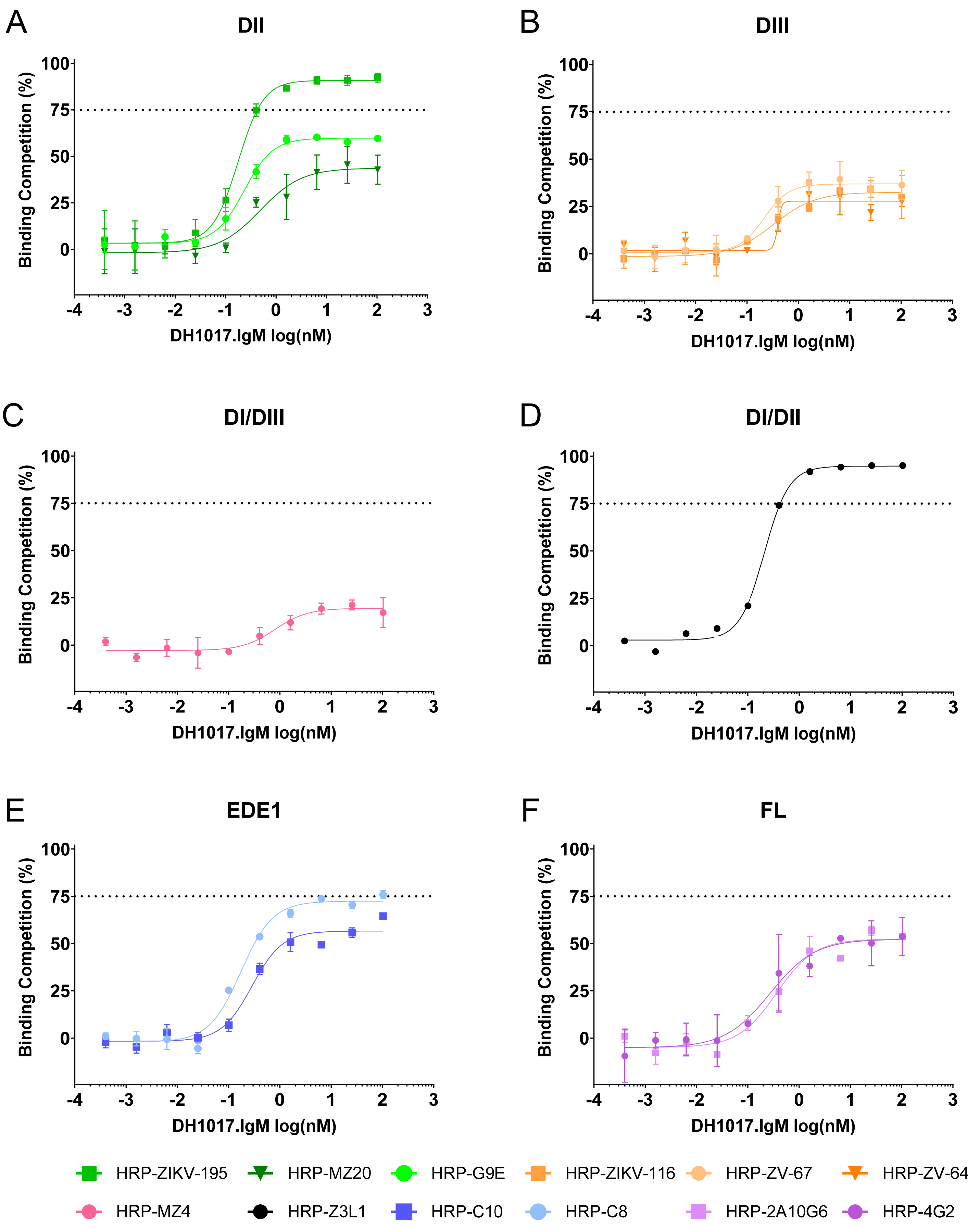

Figure S3. DH1017.IgM dose-dependent competition of ZIKV-IgG mAbs binding to ZIKV-DAK whole virion. Dose-response curves show the percentage of each of 12 ZIKV-IgG mAbs binding to ZIKV-DAK competed by DH1017.IgM grouped in panels based on IgG mAb binding regions as reported in Figure S2. Dotted lines: 75% binding reduction of each ZIKV-IgG mAb binding. Error bars: SD of two technical replicates. Data are representative of at least two independent experiments.

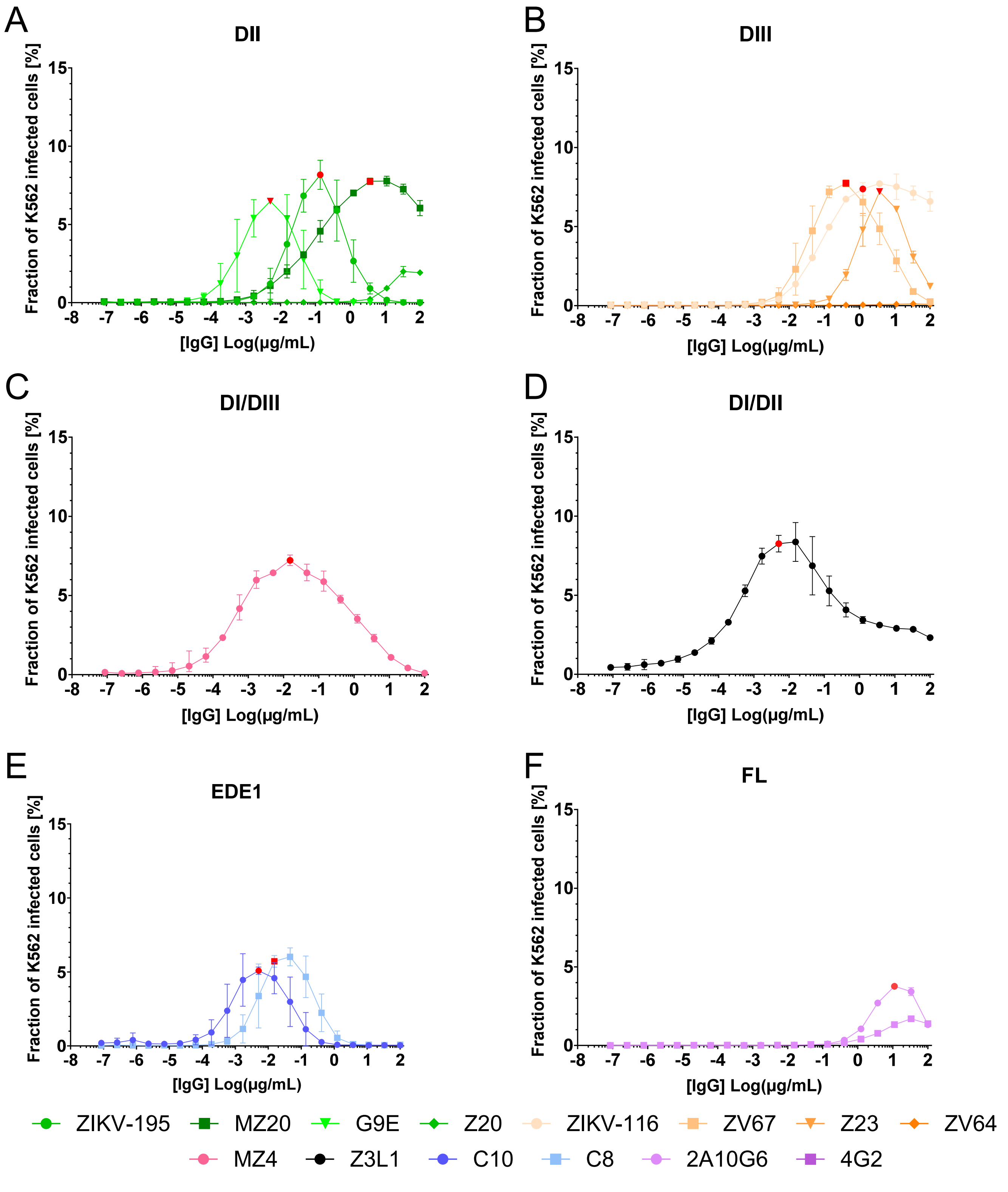

Figure S4: Antibody-dependent enhancement of infectivity (ADE) profile of the ZIKV-IgG mAbs. ADE-mediated infection of GFP-expressing ZIKV-DAK RVPs are reported for each of 14 ZIKV-IgG mAbs grouped in panels based on the respective binding regions as reported in Figure S2. The y-axis shows the percentage of infected FcγR-expressing K562 cells over the total number of acquired cells. Red dots: IgG mAb concentrations at peak ADE selected for subsequent analyses; when no clear ADE peak concentration could not be identified, the lower concentration with maximum ADE infectivity was selected. Error bars: SD of two independent replicates.

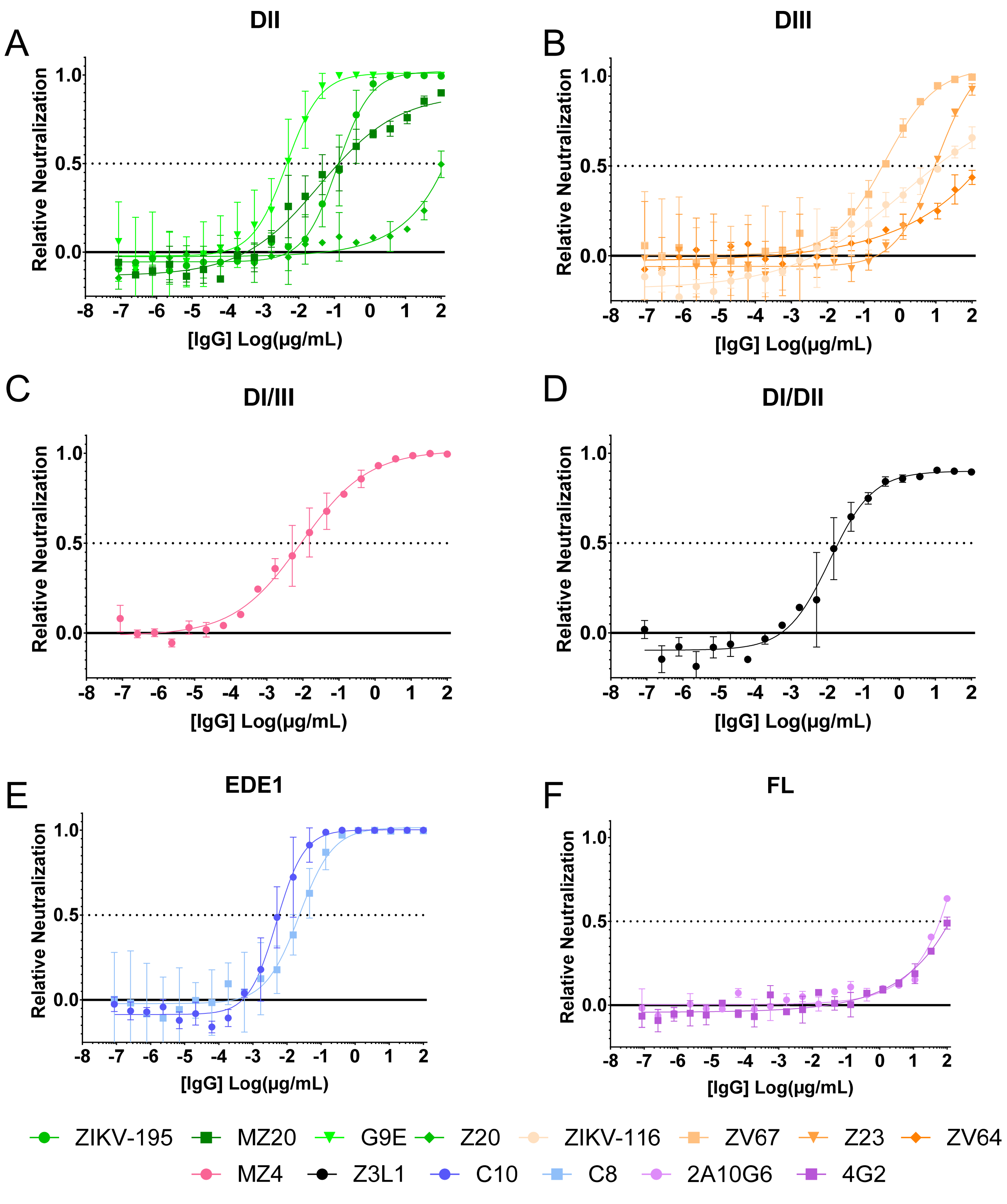

Figure S5: Neutralization profile of the ZIKV-reactive IgG mAbs. Neutralization of GFP-expressing ZIKV-DAK RVPs on Raji DC-SIGNR cells are reported for each of 14 ZIKV-IgG mAbs grouped in panels based on the respective binding regions as reported in Figure S2. The y-axis shows neutralizing activity relative to the medium-only condition. Dotted line: 50% neutralization ($RVPNC_{50}$), Error bars: SD of two independent experiments.

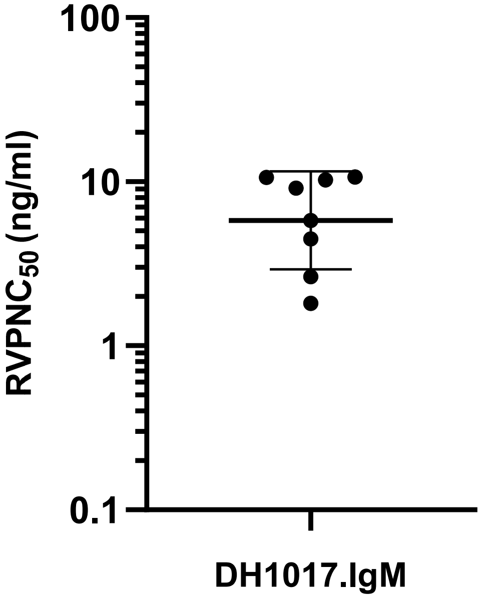

Figure S6: DH1017.IgM neutralization potency against the ZIKV-DAK RVP strain. $RVPNC_{50}$ of DH1017.IgM of GFP-expressing ZIKV-DAK RVP on Raji DC-SIGNR cells from 8 independent experiments. Line: Geometric mean; error bar: geometric SD.

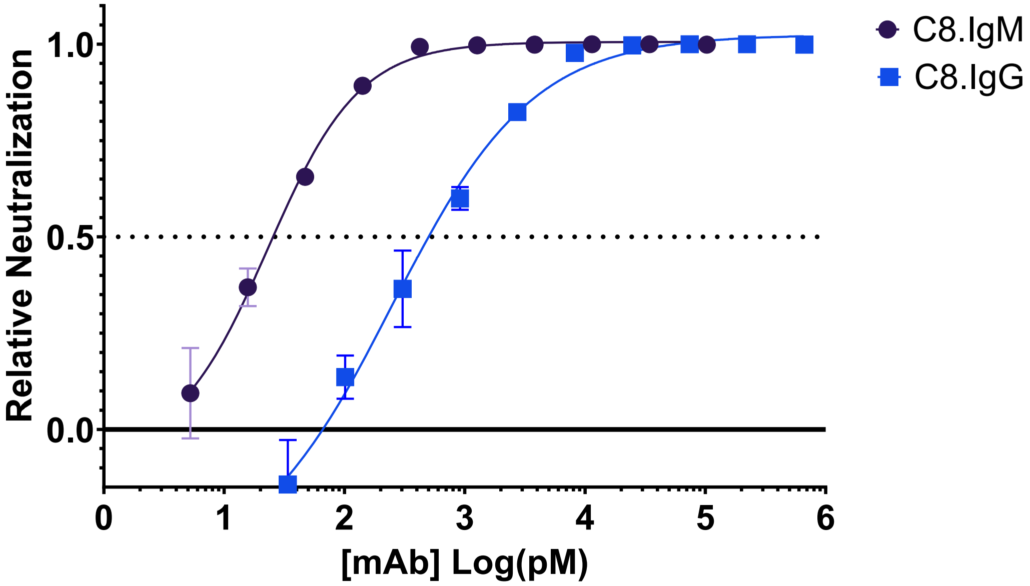

Figure S7: Neutralizing activity of C8.IgM and C8.IgG. C8.IgG (blue) and C8.IgM (black) dose-dependent neutralization of GFP-expressing ZIKV-DAK RVPs infectivity of Raji DC-SIGNR cells are reported relative to the medium-only condition. Concentrations are reported as pM with canonical molecular masses of 150 kDa and 970 kDa for the IgG and IgM isotypes, respectively. Dotted line: 50% neutralization ($RVPNC_{50}$), Error bars: SD of two independent experiments.

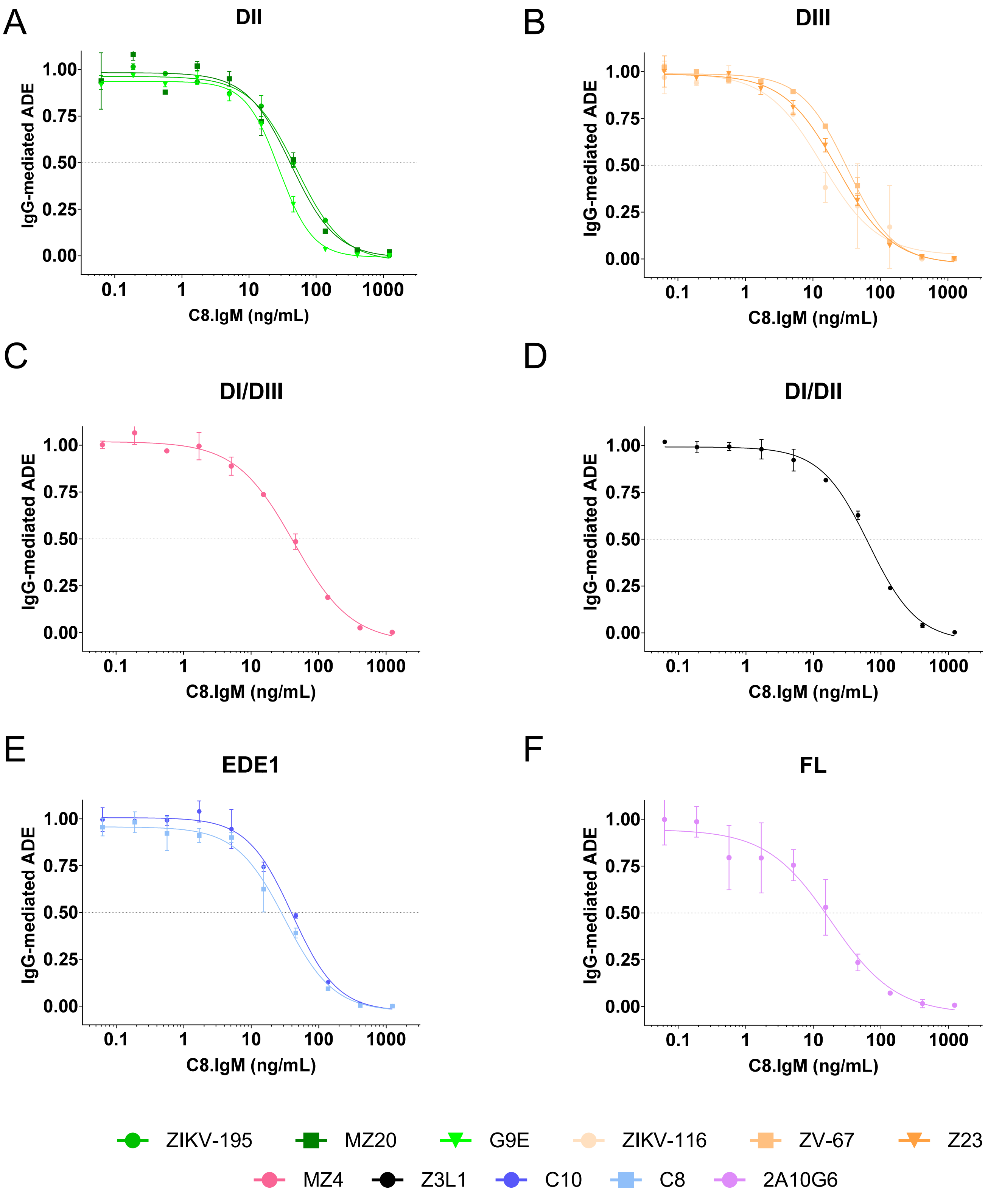

Figure S8: C8.IgM dose-dependent potency in reducing ZIKV-IgG mediated ADE infectivity. Each panel shows ADE-mediated infection of GFP-expressing ZIKV-DAK RVPs by a ZIKV-IgG mAb at their respective peak ADE concentration in presence of serially diluted C8.IgM. The y-axes show the C8.IgM dose-dependent reduction in ADE relative to the ZIKV-IgG alone condition. ZIKV-IgG mAbs are grouped in panels based on binding regions: **(A)** DII, **(B)** DIII, **(C)** DI/DIII linker, **(D)** DI/DII hinge, **(E)** EDE1, and **(F)** fusion loop (FL). Error bars: SD from two independent experiments. Dotted line: 50% reduction of ADE-infectivity (cADEC$_{50}$).
